# Supplementary material for: The Dual Prey-Inactivation Strategy of Spiders—In-Depth Venomic Analysis of Cupiennius salei
Source: Toxins (Basel). 2019 Mar 19;11(3):167. doi: 10.3390/toxins11030167 (PMC6468893; doi:10.3390/toxins11030167)
Supplement: Supplementary file 1 [file toxins-11-00167-s001.zip › Supplementary Fig S1.1-S1.19/Supplementary Fig S1.17.pdf]

Supplementary Fig S1.17

CLUSTAL O(1.2.4) multiple sequence alignment: Insulin-like growth factor-binding protein-related proteins 1

```
sp_G4V4G1_IGFBP_CUPSA      ----MWIPLLLVALVVPA--IRCERKCGECNPEKCQPPSEECLAGLVKDLCGCCYVCGRR
IGFBP_ALOMA                ----MWIPILLLALVVPS--IRSERKCDECVSEKCRPPSEECLAGLVKDLCGCCYVCGRR
IGFBP_VIRFA                ----MWIPIIVLALITPN--INGERKCGVCEPEKCQPPSEDCLAGLVKDLCGCCYVCGRR
tr_A0A2P6K9L4_NEPCL        ----MWLKLVLLTTVVVLVQAETKRKCEKCEPEKCVPPAEECLAGLVRDLCGCCYVCGRS
tr_A0A087TVX1_STEMI        ----MYFNLLILAIAFIQVRSELNDKCGECEPEKCKPPAEECLAGLVRDLCGCCYVCGRQ
tr_A0A2I9LP44_CENHE        MKWVVWLFCLL------AACVAVKRDCPPCKPQQCRPP-ENCLAGMVKDACDCCYVCARR
tr_A0A131YZ15_RHIAP        MSSIAHAT-LLLLMLLVAAASAARRECGPCDPDRCEPPRGQCLSGIVQDACGCCMVCGQR
tr_A0A0X9P0M4_9EUCA        MAKRGAFVVAAAFVVAVAVAQDVVTECGECDRSNCP-EVKTCLGGKVQDA GCCEVCALG
                                                    .*  *  ..*      **.* *:* *.** **.

sp_G4V4G1_IGFBP_CUPSA      EGELCDGDFLPIPYRNRGHGPCGEYLECRPRTDLAPGDPPEAMCVCLKTETLCGSDGKTY
IGFBP_ALOMA                EGELCDGDFLPIPYRNRGYGPCGEHLECRPRTDLAPGDPPEAMCVCLKTETLCGSDGNTY
IGFBP_VIRFA                EGELCDGDFLPIPYRNRGYGPCGENLECRPRTDLAPGDPPEAQCVCLKTETLCGSDGNTY
tr_A0A2P6K9L4_NEPCL        EGELCDGDVLPIPYRNRGHGPCGEHLECRPRTDLAPGDPPEAQCVCVKNESFCGSDGKTY
tr_A0A087TVX1_STEMI        EGELCDGDLLPIPYRNRGHGPCGEHLECRPRTDLAPGDLPEALCVCIKTEALCGNDGVTY
tr_A0A2I9LP44_CENHE        EGERCDNVSLPLPYTY-KYGYCGENLECRLRTDLLPEDPAEATCECLKQEALCASDGQTY
tr_A0A131YZ15_RHIAP        EGQRCYHRSVKG---SLADGPCGEDLECRPRRDLAPGDPAEALCVCSRPEPVCGSDGVTY
tr_A0A0X9P0M4_9EUCA        LGQRCDSQDSGD---STDYGSCGEYLVCRTRTDT--GGTDEATCVCENPDPVCGSDGITY
                            *: *              * *** * ** * *    .  ** * * . : .*..** **

sp_G4V4G1_IGFBP_CUPSA      QNECQLTEARYKQRDGLRAMHRGPCKSAPKITSPPEEASNYTGGNIAMSCEATGWPIPVF
IGFBP_ALOMA                DNECQLTEARYKQRDGLRAMHRGPCRSAPKITSPPEEASNYTGGNIAMSCEATGWPIPVF
IGFBP_VIRFA                ENECQLTEARYKQRDGLKAMHRGPCKSAPKIVTPPEEASNYTGGTIAMSCEATGWPIPIF
tr_A0A2P6K9L4_NEPCL        ENECQLTQARYTQRNGLQAVHRGPCSSGKKIFIKLN------------- ----------
tr_A0A087TVX1_STEMI        ENECQLTEARYRYRNGLQAAHRGPCKSAPKIVSPPEDVANYTGGHVALSCEAMGWPIPVV
tr_A0A2I9LP44_CENHE        DNECQLTEARYKKRDGLRAMSRGPCRSVPKIISPPEDAFNKTGGSVALSCEVSGWPIPVI
tr_A0A131YZ15_RHIAP        DNVCQLTEQRYKMRNGLEAASRGPCYTAPRVVTPPENTRNRTGGRAAMTCEVTGYPVPTV
tr_A0A0X9P0M4_9EUCA        STLCHLLQETTEKPD-VFVAVRGPCKGVPVIKSKPEDKIRPLGSILVLDCEAAGYPVPEI
                           ..  *:* :      : : .  ****     :    :

sp_G4V4G1_IGFBP_CUPSA      EWRVDIGDGNTIPLPSDDPKVSVQSRGGPSKYEVTSWLQLLSIQPKDDATYWCIAKNDEG
IGFBP_ALOMA                EWRVDIGDGNTIPLPSDDSKVSVQSRGGPNKYEVTSWLQLLSIQPKDDATYWCIAKNDEG
IGFBP_VIRFA                EWRVDRGDGDTIPLPSDDPKVSVQSRGGPSKYEVTSWLQLLSIQPKDAANYWCIAKNEEG
tr_A0A2P6K9L4_NEPCL        ---------------------------------------------------- -------
tr_A0A087TVX1_STEMI        EWRVDKGNGDTEPLPSDDPKVAVQSRGGPSRYEVTSWLQFLNINPENQATYWCIAKNELG
tr_A0A2I9LP44_CENHE        EWKVDHGNGITSTMPSDTPRISVQSRGGPRNYEVTSWLQVQRLRVQDYATYYCVARNDEG
tr_A0A131YZ15_RHIAP        EWRVDHGEGPLKALPTDSSRISVQSRGGPDSFEVTSWLQLLDLRPEDTATYWCVGANENG
tr_A0A0X9P0M4_9EUCA        TWELNRPDGSTMKLPGDDSSFAVQVRGGPEDHMVTGWVQIMRITKKSLGIYTCVATNTEG

sp_G4V4G1_IGFBP_CUPSA      ESSAAARVVVLDFRGSQTSQKGRDNDL
IGFBP_ALOMA                ESSAAARVVVLDFKGSKTEDNGRINDL
IGFBP_VIRFA                ESSAAARVVVLDFKGSNISQNERSNDL
tr_A0A2P6K9L4_NEPCL        ---------------------------
tr_A0A087TVX1_STEMI        ESSASATVKVTNLKGDHALGAERLNDL
tr_A0A2I9LP44_CENHE        ETTASARIYVTDIPRKAKLN-----EI
tr_A0A131YZ15_RHIAP        ETSAAAKLNVLP---------------
tr_A0A0X9P0M4_9EUCA        ETRVSATVALKEHGEKEDSM----NKL
```

Percent Identity Matrix - created by Clustal2.1

1: sp_G4V4G1_IGFBP_CUPSA	100.00	91.95	87.36	73.33	72.41	61.69	55.79	38.80
2: IGFBP_ALOMA	91.95	100.00	86.59	72.00	70.88	62.10	55.79	37.60
3: IGFBP_VIRFA	87.36	86.59	100.00	71.33	72.41	59.27	55.79	37.60
4: tr_A0A2P6K9L4_NEPCL	73.33	72.00	71.33	100.00	73.03	56.25	48.65	35.86

5: tr_A0A087TVX1_STEMI	72.41	70.88	72.41	73.03	100.00	56.40	53.69	38.10
6: tr_A0A2I9LP44_CENHE	61.69	62.10	59.27	56.25	56.40	100.00	55.00	38.46
7: tr_A0A131YZ15_RHIAP	55.79	55.79	55.79	48.65	53.69	55.00	100.00	38.93
8: tr_A0A0X9P0M4_9EUCA	38.80	37.60	37.60	35.86	38.10	38.46	38.93	100.00

Proteomic coverage trypsin

>sp|G4V4G1|IGFBP_CUPSA Insulin-like growth factor-binding protein-related protein 1 OS=Cupiennius salei OX=6928 PE=2 SV=1

MWIPLLLVAL VVPAIRCERK CGECNPEKCQ PPSEECLAGL VK**DLCGCCYV CGR**REGELCD GDFLPIPYRN RGHGPCGEYL
ECRPRTDLAP GDPPEAMCVC LKTETLCGSD GKTYQNECQL TEARYKQRDG LRAMHRGPCK SAPKITSPPE EASNYTGGNI
AMSCEATGWP IPVFEWRVDI GDGNTIPLPS DDPKVSVQSR **GGPSKYEVTS WLQLLSIQPK** DDATYWCIAK NDEGESSAAA
RVVVLDFRGS QTSQKGRDND L

Proteomic coverage chymotrypsin

Supplementary Fig S1.17 Multiple sequence alignment of IGFBP-rPs from *C. salei* (sp_G4V4G1_IGFBP_CUPSA), *A. marikovskyi* (IGFBP_ALOMA), *V. fasciatus* (IGFBP_VIRFA), *N. clavipes* (tr_A0A2P6K9L4_NEPC), *S. mimosarum* (tr_A0A087TVX1_STEMI), the scorpion *Centruroides hentzi* (tr_A0A2I9LP44_CENHE), the tick *Rhipicephalus appendiculatus* (tr_A0A131YZ15_RHIAP), and the green rock lobster *Sagmariasus verreauxi* (tr_A0A0X9P0M4_9EUCA). Signal peptides are marked in black characters, cysteines in white with a black box, positively charged amino acids in pink, negatively charged amino acids in blue, hydrophobic amino acids in red and polar amino acids in green. The catalytic triad is highlighted with gray, the involved amino acid residues of the proposed active site binding structure are highlighted in yellow. The processing site to obtain the proposed activated serine protease is highlighted in red and the Gly that is used for C-terminal amidation of the light chain is highlighted in blue.
